# Anti-Oxidative Bioactivities of Medicinal Herbs in the Treatment of Aging-Related Diseases

**DOI:** 10.3390/antiox13060631

**Published:** 2024-05-22

**Authors:** Hye-Sun Lim, Gunhyuk Park, Yong-Ung Kim

**Affiliations:** 1 Herbal Medicine Resources Research Center, Korea Institute of Oriental Medicine, Daejeon 58245, Republic of Korea; qp1015@kiom.re.kr; 2 Korean Convergence Medicine Major, Campus of Korea Institute of Oriental Medicine, University of Science & Technology (UST), Daejeon 34113, Republic of Korea; 3 Department of Pharmaceutical Engineering, College of Herbal Bio-Industry, Daegu Haany University, Gyeongsan 38610, Republic of Korea

Over the last 20 years, significant progress has been made in understanding the biology of aging and lifespans. Previous studies have reported that lifespan and aging rates in various animal species can be genetically modified and that targeting specific characteristics of aging using certain molecules or environmental interventions can delay the onset of, or prevent the development of, several diseases, as well as stimulating tissue and organism rejuvenation. As the human lifespan extends, age-related diseases are undergoing a corresponding evolution, prompting researchers within the scientific and technological communities to focus on prevention and treatment methods.

Although genetics has long been considered a primary factor in aging and age-related diseases, it is now understood that genetic factors alone cannot fully account for differences in the aging process. As identical twins do not always age at the same rate or have the same lifespan, external factors must play a role. Since the 1950s, the leading hypothesis in aging research has been the oxidative damage theory, which is rooted in reactive oxygen species (ROSs), commonly known as free radicals [1]. Oxygen, which is essential for energy production, generates a small fraction of highly reactive free radicals during cellular metabolism [1,2]. These radicals, which lack one electron and are highly unstable, readily engage in oxidative reactions with molecules such as cell membranes, DNA, and lipids, leading to cellular damage and, ultimately, contributing to various diseases and to aging [3].

The body inevitably produces ROSs as part of normal physiological processes, including immune responses against invading pathogens [3]. While ROSs play essential roles in defense mechanisms, their excessive production can result in oxidative stress, which damages normal tissues and exacerbates disease processes [4]. Approximately 90% of modern human diseases are associated with oxidative stress [5]. ROSs can be generated in any organ or tissue, and their detrimental effects are linked to degenerative diseases, such as cardiovascular diseases, dementia, arthritis, and cataracts. They also contribute to aging [5].

Therefore, in this Special Issue, we explore the potential of various medicinal herbs in terms of their ability to suppress the oxidative damage caused by ROSs. Our aim is to showcase research findings regarding treatments for age-related diseases, offering valuable insights to scientists and clinicians.

Gurung et al. report that aging-related chronic inflammation impairs memory-related molecules such as BDNF/ERK/CREB by increasing ROSs and pro-inflammatory mediators such as NF-κB, TNF-α, and COX-2 and lowering IL-10 levels in the hippocampus (Contribution 1). These cognitive abnormalities were reversed using *Euonymus alatus* leaf extract in aged mice and scopolamine-induced neuroblastoma cells. *E. alatus* leaf extract had similar effects in LPS-induced neuroblastoma and microglial cells. Therefore, the antioxidant and anti-inflammatory effects of *E. alatus* leaf extract are protective and may improve cognitive function during normal aging. This treatment also enhanced neurogenesis, suggesting that *E. alatus* leaf extract has potential therapeutic value in aging and aging-related conditions.

Alzheimer’s disease (AD) is caused by a variety of mechanisms; therefore, treatments should have multiple functions. Jadhav et al. demonstrated the effects of the combined administration of memantine, which is mainly used in clinical practice, and baicalein, which has recently shown excellent therapeutic effects for the treatment of AD (Contribution 2). This therapy has anti-oxidant, AChE-inhibitory, memory- and learning-enhancing, Aβ-plaque-reducing, and BDNF-expression-enhancing effects, resulting in a combined neuroprotective effect against Aβ-induced AD in albino Wistar rats.

Balakrishnan et al. successfully synthesized AD-1, a new drug based on a-asarone and confirmed its efficacy against various neurodegenerative diseases (Contribution 3). AD-1 has been reported as effective for short-term memory loss and has significantly lowered the levels of apoptotic markers and increased neuronal survival. Furthermore, AD-1 ameliorated scopolamine-induced impairments in spatial learning behavior and memory formation via the regulation of NF-κB/MAPK signaling. Therefore, this drug may be a promising candidate for use in the development of treatments for neurodegenerative disorders such as AD.

Kim et al. have presented medicinal herbs with excellent anti-inflammatory effects in several previous studies, and they report a new composition in this Special Issue (Contribution 4). They have discovered an efficient ratio of Prunella vulgaris and Gentiana lutea that yields better inflammatory inhibitory effects than each compound alone. This combination has a stronger anti-inflammatory effect, suppressing the destruction of artificial cartridge, and may play an important role in the development of arthritis-inhibitory drugs in the future.

Liang et al. studied biomarkers for the mechanisms of various pathways in AD and investigated the use of phenotypic screening analysis to identify compounds with therapeutic efficacy for AD (Contribution 5). Aging, cognition, and AD pathological target pathways that reflect neurotoxic pathways by age, rather than single molecular targets, were analyzed. The use of a robust battery of novel, cell-based phenotypic screening assays allowed for the comparison of sterubin from Yerba santa (Eriodictyon californicum) with seven structurally closely related flavonoids through identifying the specific combination of modifications to the hydroxyl/methoxy ring, phenol ring, and C2–C3 bond ring in sterubin. This minor structural change led to a unique compound that is highly protective against multiple insults related to aging and AD and has strong anti-inflammatory activity. From a medicinal chemistry perspective, these results further emphasize how very small structural changes in flavonoids can dramatically affect their activities against distinct insults. Structure–activity relationship data provide additional support and mechanistic guidance for the further development of sterubin as a potential neuroprotective drug candidate.

Pérez et al. define the mechanism and target of neurodegenerative diseases after studying the antioxidant mechanism based on Nrf2 and NFkB control (Contribution 6). Data collected over the last 13 years related to the caffeic acid phenethyl ester, which has potential as a therapeutic agent, are analyzed and reviewed. Thus, several researchers’ works could have a profound impact on the design of treatment strategies for various cranial nervous system diseases, such as dementia and Parkinson’s disease.

In addition, Qiao et al. define various risks based on aging and attempt to analyze treatment strategies using natural products (Contribution 7). Important information is provided for researchers and industries on the identification of the points, strengths, and weaknesses that should be considered when developing a natural-product-based treatment.

Globally, several researchers are analyzing the relationship between aging and disease and promoting various strategies to overcome aging. This Special Issue was designed to focus on the effects of suppressing oxidative stress using natural products and the development of treatments, and we are confident that this Special Issue will inspire more researchers.

## References

[1] Golden T., Hinerfeld D.A., Melov S. (2002). Oxidative stress and aging: Beyond correlation. Aging Cell.

[2] Detienne G., Haes W., Mergan L., Edwards S., Temmerman L., Bael S. (2018). Beyond ROS clearance: Peroxiredoxins in stress signaling and aging. Ageing Res. Rev..

[3] Brown G. (2015). Living too long. EMBO Rep..

[4] Garmany A., Yamada S., Terzic S. (2021). Longevity leap: Mind the healthspan gap. NPJ Regen. Med..

[5] Jen A., Samal R., Bhol N., Duttaroy A. (2023). Cellular Red-Ox system in health and disease: The latest update. Biomed. Pharmacother..

